# Adapting a mobile TB screening unit to provide integrated screening services and linkage to primary care

**DOI:** 10.5588/pha.24.0025

**Published:** 2024-12-01

**Authors:** A.K. Millones, S. Cohen, D. Acosta, H. Campos, A. Condeso, S. Farroñay, I. Torres, C. Vargas, K. Abanto, C. Contreras, S. Palomino, J. Jimenez, L. Lecca, C.M. Yuen

**Affiliations:** ^1^ Socios En Salud Sucursal Peru, Lima, Peru;; ^2^ Department of Medicine, Brigham and Women’s Hospital, Boston, MA, USA;; ^3^ Department of Global Health and Social Medicine, Harvard Medical School, Boston, MA, USA;; ^4^ School of Social Work, University of South Florida, Tampa, FL, USA.

**Keywords:** community health services, community health workers, patient navigation, mass screening, tuberculosis

## Abstract

**OBJECTIVE:**

We adapted a mobile TB screening unit to create an integrated screening program for noncommunicable diseases and TB, using community health worker (CHW) navigators to support linkage to care. We piloted the model in underserved communities of Lima, Peru, evaluating its feasibility, acceptability, and ability to continue supporting TB case detection.

**DESIGN:**

The program provided screening for TB, hypertension, diabetes, and depression and was rebranded to avoid TB-associated stigma. CHW navigators were trained to link people to care for all four conditions. Implementation barriers and facilitators were identified from the implementation team’s meeting minutes.

**RESULTS:**

During August–December 2023, we screened 1,000 adults, of whom 254 (25%) were referred for evaluation and paired with CHW navigators. Of these, 197 (78%) underwent evaluation at a health center, and 151 (59%) initiated some form of treatment, including 4 for TB. Completion of the linkage cascade was 93% for TB, 81% for hypertension, 71% for diabetes and 69% for depression. Limitations in equipment and staff were implementation barriers, while multiple flexibility-related facilitators were identified.

**CONCLUSION:**

The integrated screening program was acceptable and feasible and still identified people with TB. CHW navigators were effective in supporting linkage to primary care services.

## Abstract (fr)

**OBJECTIF:**

Nous avons adapté une unité mobile dédiée au dépistage de la TB afin de mettre en place un programme intégré de dépistage des maladies non transmissibles et de la TB. Pour ce faire, nous avons mobilisé des agents de santé communautaires (CHW) afin d'améliorer l'accès aux soins. Ce modèle a été testé dans des communautés défavorisées de Lima, au Pérou, dans le but d'évaluer sa faisabilité, son acceptabilité et sa capacité à maintenir le soutien à la détection des cas de TB.

**MÉTHODES:**

Le programme a permis le dépistage de la TB, de l'hypertension, du diabète et de la dépression et a été renommé afin de réduire la stigmatisation liée à la TB. Les CHW ont reçu une formation pour orienter les individus vers les soins appropriés pour ces quatre pathologies. Les défis et les éléments favorables à la mise en œuvre ont été déterminés à partir des comptes rendus des réunions de l'équipe chargée de l'exécution.

**RÉSULTATS:**

Entre août et décembre 2023, nous avons dépisté 1 000 adultes, parmi lesquels 254 (25%) ont été dirigés vers une évaluation et mis en contact avec des navigateurs du CHW. Parmi ces personnes, 197 (78%) ont reçu une évaluation dans un centre de santé et 151 (59%) ont commencé un traitement, dont 4 pour la TB. Le taux d'achèvement de la cascade de liaison s'élevait à 93% pour la TB, 81% pour l'hypertension, 71% pour le diabète et 69% pour la dépression. Les limitations en matière d'équipement et de personnel ont représenté des obstacles à la mise en œuvre, tandis que plusieurs facilitateurs liés à la flexibilité ont été identifiés.

**CONCLUSION:**

Le programme de dépistage intégré s'est avéré satisfaisant et réalisable, facilitant l'identification des individus atteints de TB. Les CHWs ont joué un rôle efficace dans le maintien du lien avec les services de soins primaires.

Community-based screening programs are an established method to improve the detection of various health conditions, including infectious diseases^1,2^ and noncommunicable diseases (NCDs).^3,4^ Designed to reach populations that face barriers to accessing health services, these programs allow individuals to reach services at easy-to-access sites within the community. However, many programs in low- and middle-income countries (LMICs) focus only on one condition, which has limitations. Uptake may be limited if the condition is stigmatized or awareness in the community is low. In the case of a relatively rare condition such as TB, substantial resources are invested to find a small number of cases. At the same time, the vast majority of people screened will not benefit from any medical care. Finally, these programs often rely on condition-specific donor funding, threatening sustainability.

` Existing community-based TB screening platforms present an opportunity for broader delivery of primary care screening services in communities. Common NCDs, including physical NCDs such as hypertension, as well as mental NCDs such as depression – are underdiagnosed and undertreated in LMICs^5,6^ and easy to screen for with limited medical equipment. Combining TB and NCD screening could destigmatize TB screening by making it part of a general health program. However, linking people to NCD care may be more challenging given their prevalence, lack of disease-specific donor support, and lower prioritization by many health systems. Also, it is unclear whether a general screening program that deemphasizes TB would continue to be effective for TB case-finding if the people attracted to the program are at lower risk for TB.

Many programs have reported screenings for physical and mental NCDs (e.g. diabetes mellitus [DM], hypertension, depression) among people with TB or their contacts or TB screenings for people receiving care for NCDs.^7,8^ However, these approaches only reach people already receiving care for one condition. In contrast, we adapted a mobile TB screening unit in Lima, Peru, to create an integrated community-based screening program for NCDs and TB that sought to broadly reach people disconnected from the healthcare system, using trained community health worker (CHW) navigators to promote linkage to care for multiple conditions.

## STUDY POPULATION, DESIGN, AND METHODS

We conducted a pilot to evaluate the feasibility and acceptability of the integrated community-based screening program and its ability to continue supporting TB case detection. There was no planned comparison between the integrated and original TB-specific screening programs.

### Study population and setting

The intervention was implemented from August to December 2023 by the non-governmental organization Socios En Salud (SES) in the Carabayllo District of Lima, Peru. Carabayllo had a population of approximately 333,000, with 23% poverty in the 2017 census.^9^ Peru has an estimated TB incidence of 150/100,000 population,^10^ estimated DM prevalence of 8% in Lima,^11^ and estimated hypertension prevalence of 20% nationally;^12^ in a 2018 national survey, 2% of adults in Lima reported moderately severe or severe depressive symptoms.^13^

### Intervention

Since 2019, SES has implemented a community-based TB screening program using mobile screening units with chest radiography.^14^ For this intervention, we adapted the mobile units to provide screening for TB, hypertension, DM, and depression, and we rebranded them as integrated health screening units to avoid TB-associated stigma. The adaptation was based on the results of a preference survey in which people with TB suggested that they would have attended this type of integrated screening program and, hence, been diagnosed more promptly.^15^ SES partnered with local community leaders who helped identify screening locations in underserved areas where residents found it difficult to access health centers. Community leaders informed residents about the program and encouraged attendance. People ≥18 years old were eligible for participation. All participants were expected to complete screening for all four conditions unless they reported a prior diagnosis of hypertension, DM, or current TB (in which case they were not screened for that condition). Screening procedures and referral criteria are described in Table 1.

**TABLE 1.** Screening and referral procedures.

Condition	Screening procedure	Positive screen criteria for referral	Procedures after referral
TB	Chest radiography was performed using a portable X-ray machine equipped with CAD4TB V.6 software (Delft Imaging, ’s-Hertogenbosch, Netherlands). Participants with a CAD4TB abnormality score >50 were evaluated by a physician at the screening unit and submitted a sputum sample for Xpert^®^ MTB/RIF Ultra testing (Cepheid, Sunnyvale, CA)	Positive GeneXpert result or physician’s clinical suspicion	Evaluation for TB at participant’s primary care center
Hypertension	Blood pressure was taken with an automatic blood pressure cuff after participants had been seated for 5 minutes. Participants with systolic pressure ≥140 mmHg or diastolic pressure ≥90 mmHg had measurement repeated	Average systolic pressure ≥140 mmHg or average diastolic pressure ≥90 mmHg over two measurements	1 week of daily blood pressure measurements, then evaluation at primary care center if ≥1 measurement over 140/90 mmHg
Diabetes	A point-of-care finger-stick glycosylated hemoglobin (HbA1c) test was performed using the Afinion 2 Rapid Diagnostic system (Abbott, Abbott Park, IL, USA)	HbA1c ≥6.5%	Glucose testing performed at the primary care center laboratory, followed by clinical evaluation
Depression	The 9-question Patient Health Questionnaire (PHQ-9) depression screening instrument was administered	PHQ-9 score ≥15 (moderately severe or severe depression) or suicidal ideation	Evaluation by a psychologist at a primary care center or community mental health center

All participants received counselling about their screening results, and those meeting the criteria were referred to the public health system and paired with a trained CHW navigator. CHW navigators helped participants make appointments at health centers, made reminder phone calls, and often accompanied participants to appointments and advocated for them. CHW navigators were trained to conduct blood pressure measurements in participants’ homes as an initial evaluation step following a positive hypertension screen. If CHW navigators identified participants who required socioeconomic assistance to complete evaluations, they connected them to a social protection team at SES, which provided additional support, such as help registering for health insurance and subsidies for transportation to health centers. CHW navigators were supposed to accompany participants for 2 months following the initial screening, although in practice, they sometimes remained engaged for longer; frequency of contact and type of support was individualized to participants’ preferences.

### Feasibility and acceptability of data collection and analysis

Feasibility was assessed by evaluating the screening and linkage cascades for each condition. Data on screening procedures was collected in real time by staff implementing the intervention. To assess the linkage cascades, we asked CHW navigators to prospectively report whether participants were evaluated at a health center, the date of the evaluation, and the results of the evaluation (as reported by the participants and verified at the health center). Among those referred to care following a positive screen, we calculated the percentage who completed the linkage cascade, defined as either initiating treatment or being determined not to require treatment.

To assess acceptability among participants, we administered the 8-item Client Satisfaction Questionnaire (CSQ-8) to all participants at the end of the screening event, asking about their satisfaction with the screening services. We attempted to contact all participants referred for evaluation 2–3 months after the screening to administer a second CSQ-8 focused on satisfaction with the CHW navigators’ support. At this time, we also asked participants whether they were taking treatment for any of the four conditions of interest and an open-ended question about suggestions for improvement of the program.

We identified barriers and facilitators to implementation based on the implementation team’s meeting minutes. During weekly meetings, field staff were asked to identify the obstacles and facilitators to implementation that they experienced or observed. The written minutes from these meetings were later subjected to a deductive coding process (performed in Microsoft Excel), in which barriers and facilitators were categorized based on a patient-centered framework that conceptualizes access to health care according to 5 domains (approachability, acceptability, availability and accommodation, affordability, and appropriateness), which capture aspects of both feasibility and acceptability.^16^ Themes were extracted into matrices for interpretation.

### Ethical approval

The study protocol was approved by Institutional Review Boards at Mass General Brigham and Socios En Salud. All participants who attended the screening unit were enrolled with written informed consent.

## RESULTS

### Care cascades for screening and linkage to care

We enrolled 1,000 participants at 29 screening events. Participants had a median age of 46 years (interquartile range [IQR] 35–60), and 68% were female. Table 2 summarizes screening and linkage cascades, with details provided in the Figure for TB and Supplementary Figures S1–S3 for other conditions. Three (<1%) participants reported currently receiving TB treatment, 57 (6%) reported a prior diagnosis of DM, and 98 (10%) reported a previous diagnosis of hypertension. After excluding those with existing diagnoses, 254 (25%) participants screened positive for at least one condition and were referred for further evaluation, of whom 25 (10%) were referred for more than one condition. Following screening, 14 (1%) were referred for possible TB, 42 (5%) for a positive hypertension screen, 55 (6%) for a positive DM screen, 121 (12%) for moderately severe or severe depressive symptoms, and 50 (5%) for suicidal ideation with less severe depressive symptoms.

**TABLE 2.** Screening and linkage cascade for attendees of community screening events (*n* = 1,000).*

Numerator/denominator	TB *n/N* (%)	Hypertension *n/N* (%)	Diabetes *n/N* (%)	Depression *n/N* (%)
Self-reported existing diagnosis/all participants	3/1,000 (<1)	98/1,000 (10)	57/1,000 (6)	Not assessed
Screened/eligible	951/997 (95)	902/902 (100)	942/943 (>99)	981/1,000 (98)
Referred/screened	14/951 (1)	42/902 (5)	55/942 (6)	171/981 (17)
Completed initial follow-up evaluation^†^/referred	14/14 (100)	39/42 (93)	43/55 (78)	133/171 (78)
Completed full evaluation/completed initial evaluation	13/14 (93)	34/39 (87)	38/43 (88)	Not applicable
Treatment indicated for specified condition^‡^/completed full evaluation	4/13 (31)	21/34 (62)	13/38 (34)	128/133 (96)
Initiated treatment/treatment indicated	4/4 (100)	21/21 (100)	13/13 (100)	112/128 (88)

* Results are presented by condition, so individuals with multiple conditions are represented in different columns.

† For TB, hypertension, and diabetes, the initial follow-up evaluation was a clinical or laboratory evaluation at the health center. The initial follow-up evaluation for hypertension was a week of blood pressure measurements performed at home.

‡ People who started only cholesterol or pre-diabetes treatment following hypertension or diabetes evaluation are not included in the numerator or the next step of the cascade. For depression, 16 people completed the evaluation and did not initiate treatment, but we do not know whether or not treatment was indicated. We have conservatively assumed that they had treatment indicated for this analysis.

**FIGURE.**
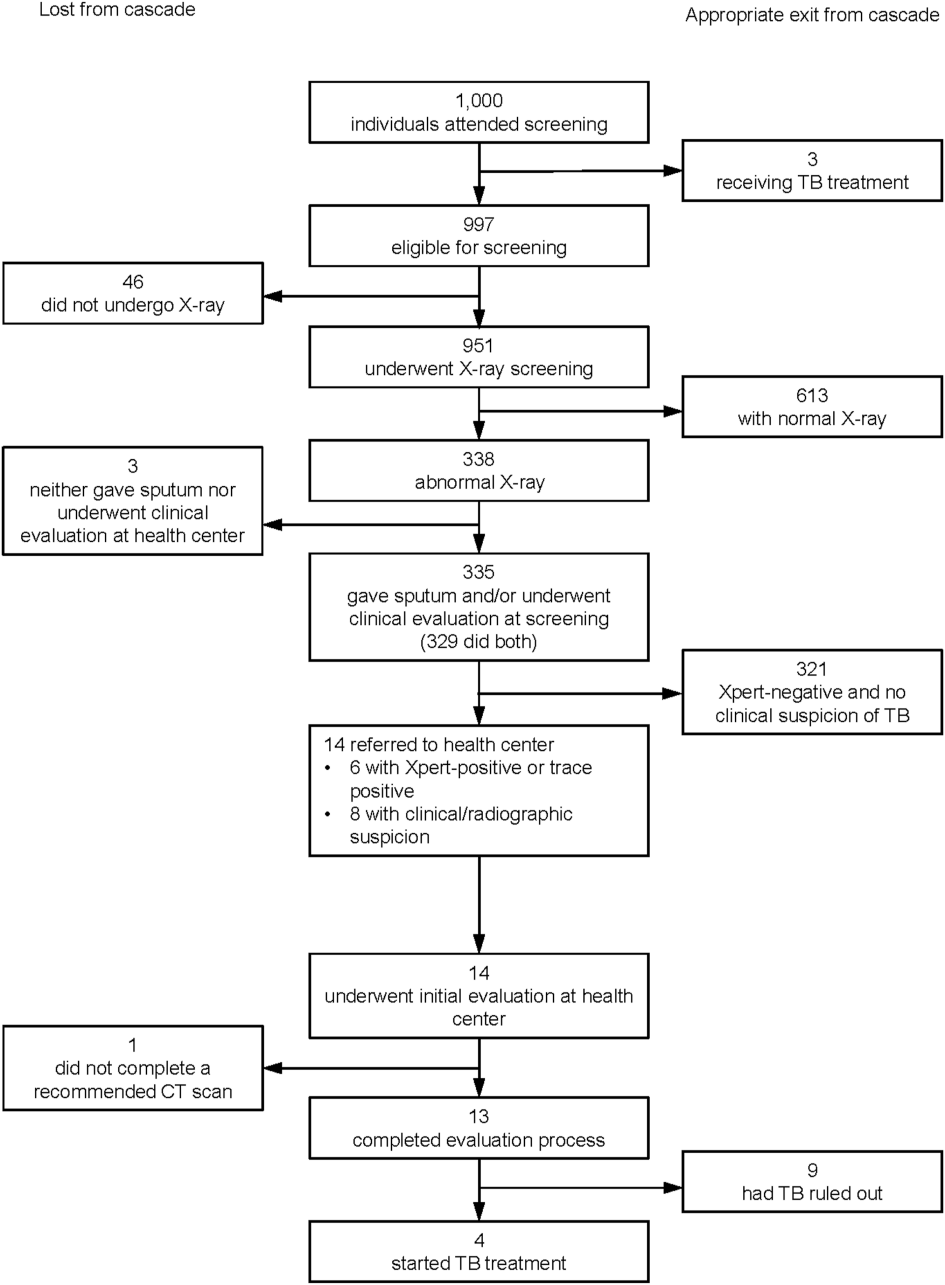
Screening and linkage cascade for TB.

Of the 254 referred, 197 (78%) underwent evaluation at a health center, and 151 (59%) initiated some form of treatment, including four treated for TB. The median time to first health center evaluation was 12 days (IQR 6–21). Among those referred, 93% completed the linkage cascade for TB, 81% for hypertension, 71% for DM and 69% for depression. The final survey was completed by 223 (88%) participants referred for follow-up, a median of 12 weeks (IQR 9–15) after initial screening. Among those who responded to the survey and who had initiated some form of treatment, 100% (4/4) were still taking TB treatment, 65% (13/20) were still taking hypertension treatment, 67% (8/12) were still taking DM treatment, 65% (11/17) were still taking depression medication, and 25% (22/87) were still receiving psychotherapy at the time of the survey.

### Acceptability among recipients

The median CSQ-8 score among 992 individuals who completed the post-screening survey was 31 (IQR 30–32) out of 32. The median CSQ-8 score for the linkage process among those who completed the final survey was 29 (IQR 25–32) out of 32. In response to the open-ended question about program improvement, 30 (13%) suggested that the screening events include more health conditions, with dental and ophthalmological care frequently mentioned, and 21 (9%) desired extended accompaniment by the CHW navigator.

### Implementation barriers and facilitators

Table 3 shows barriers and facilitators to screening and linkage to care identified at staff meetings in different domains of health care access (further description in Supplementary Data). The domain most frequently discussed was availability and accommodation, in which equipment and staff limitations impacted various implementation aspects. Limitations in equipment and trained counselors led to delays during screening, while provider shortages and lack of laboratory capacity in health centers made linkage to care less convenient and timely. Concerns about equity arose regarding approachability (i.e. which community members were aware of the campaigns) and availability (i.e. not helping people with prior diagnoses due to capacity limitations). While the screening was generally acceptable and affordable, participants encountered barriers in these domains during linkage to care.

**TABLE 3.** Perceived barriers and facilitators to implementing screening and linkage to care reported by the intervention team during weekly meetings.

Domain	Screening	Linkage to care
Approachability (Can people identify the services they need?)	Barriers: Equitable appointment distribution	Barriers: Memory issues in older participants Lack of cell phone access
	Facilitators: Broader population reached compared to TB-only screening	Facilitators: Involvement of participants’ families
Acceptability (Do people accept services?)	Barriers: Mental health stigma	Barriers: Lower acceptability of mental health care TB stigma Some people do not want to be contacted by CHWs
	Facilitators: Requiring screening for everyone reduces mental health stigma Clear expectations set about the duration of screening	Facilitators: Participants determine the level of desired support
Availability and accommodation (What services are available, and how convenient and timely are they?)	Barriers: Bottlenecks at point-of-care machines Lengthy depression screening Limited trained counselors Greater demand than capacity meant not helping people with prior diagnoses	Barriers: Difficulty getting to the health center Provider shortages lengthen wait times Lack of laboratory supplies and capacity Inflexible health system protocols and non-integrated care Complex diagnostic protocols require multiple appointments
	Facilitators: Delays reduced by flexibility in the order of screenings CHWs help direct participants through screening stations Flexible personnel allocation improves flow Wider geographic reach than prior campaigns in the same district	Facilitators: Invested providers Prior coordination with health centers Contacts within health system expedite appointments Health centers with their own laboratories have shorter delays Laboratory supply donations increase available services
Affordability (How much time and resources are spent accessing services?)		Barriers: Opportunity cost from missing work Insurance issues
		Facilitators: Support for insurance enrollment SES social protection team support
Appropriateness (How responsive are services to people’s needs?)	Barriers: PHQ-9 misses other mental health problems	Barriers: Dissatisfaction with treatment by staff at the health center CHWs dedicated to single health conditions
	Facilitators: Counseling about where to access mental health care	Facilitators: Diagnosis of other health conditions Access to TB experts Increased patient understanding of their health CHWs trained to be multi-disease health system navigators

CHW = community health worker.

Facilitators related to flexibility occurred in multiple domains. Flexibility in participant flow and personnel allocation reduced delays at the screening event, while responsiveness to the participants’ individual preferences about their desired level of support increased the acceptability of the CHW navigators. Conversely, a lack of flexibility in requiring all participants to undergo all procedures helped to increase acceptability by reducing the stigma around mental health screening. Collaboration with health system staff was also an important facilitator for improving the availability of services, as pre-booking appointment times before screenings and utilizing contacts within the health system to expedite appointment scheduling helped promote prompt attention.

## DISCUSSION

We demonstrate the feasibility of expanding a community-based TB screening platform to address common NCDs and the effectiveness of CHW navigators in supporting linkage to care for multiple conditions. This program was highly accepted in underserved communities in Lima, Peru. While accommodating lower screening volumes than the TB-only program due to the additional procedures, the integrated program still diagnosed people with TB (400 per 100,000 screened), and it connected a much larger proportion (15%) of attendees to care given that the NCDs were much more common than TB. With the support of CHW navigators, the majority of people referred after screening were successfully linked to care in the public health system. However, more support may be required to improve retention on NCD treatment after initial linkage to care.

Since community-based screening programs are often used to reach people who face barriers to accessing the health system, poor linkage to care can occur if people are not supported after screening.^17–20^ Our program provides an example of CHWs trained to navigate a complex health system where care for different conditions is not integrated and involves separate, rigid diagnostic protocols. However, we observed high proportions of people disengaging from DM, hypertension, and depression care within a few months after successful linkage. While the TB program in Peru provides support and follow-up to encourage treatment adherence, similar support was not available to people with NCDs. This may explain our anecdotal observation that many participants who reported prior DM or hypertension diagnoses mentioned that they were not currently on treatment. The acceptability of the CHW navigators supports the adoption of CHW-led strategies that have improved hypertension management,^21^ mental health,^22^ and DM management in LMICs,^23^ and strategies to promote awareness and knowledge about NCDs within communities.

The 12% prevalence of moderately severe or severe depressive symptoms among participants far exceeded the 2% found in a 2018 national survey that used the same screening instrument.^13^ This difference could reflect the demographics of attendees, which overrepresented groups with a higher prevalence of depressive symptoms (e.g. women, older adults, and people with less wealth). It could also reflect the lingering impact of the COVID-19 pandemic,^24^ or the program attracting people with unmet mental health care needs given the promise of support in accessing care. While the majority of those referred saw a psychologist in a primary care or community mental health center, delays in obtaining appointments and the high demand for services underscore the need for scalable community-based approaches to mental health care in this setting.

Our integrated screening program aligns with policy initiatives to integrate TB into general primary care services and Universal Health Coverage policies.^25^ Because communities facing healthcare access barriers likely have unmet needs for TB and NCD services, it could be efficient for health systems to adapt and expand existing care delivery platforms for TB programs to address gaps in diagnosing and treating NCDs. Notably, our program extends beyond focusing on people who have both TB and another comorbid condition (e.g. HIV, DM), an area of integration in which there have been considerably more published examples.^7,8,25,26^ Lessons from these more focused experiences apply to broader integration, including the importance of collaborative financing and budgeting, reorienting disease-focused models of care to person-centered models, and training a multidisciplinary workforce.^25^

Our evaluation had important limitations. We lacked the resources to perform qualitative research through interviews. As a result, our assessment of acceptability was limited, and the CSQ-8 results are subject to social desirability bias, given that intervention staff administered the survey. Moreover, our identification of barriers and facilitators to feasibility was limited to the observations of implementation staff, which does not capture larger health system and policy-related barriers such as disease-specific funding streams and additional program costs related to NCD services. Finally, while enabling us to study feasibility in a programmatic context, our pragmatic data collection approach did not allow us to understand whether this integrated care model reaches individuals who would not otherwise have attended a TB screening event.

In conclusion, we expanded a community-based TB screening program into one that addressed TB and common NCDs, demonstrating high feasibility and acceptability for the integrated care model. Support from trained CHW navigators was important for achieving high linkage to care across multiple health conditions. This program offers a model for providing patient-centered screening services and linkage to primary care, supporting TB case detection while more broadly addressing unmet healthcare needs in underserved communities.

## Supplementary Material


