# Supplementary material for: Adapting a mobile TB screening unit to provide integrated screening services and linkage to primary care
Source: Public Health Action. 2024 Dec 1;14(4):169–74. doi: 10.5588/pha.24.0025 (PMC11604155; doi:10.5588/pha.24.0025)
Supplement: Supplementary file 1 [file pha24-0025_supplementarydata1.pdf]

http://dx.doi.org/10.5588/pha.24.0025

Adapting a mobile TB screening unit to provide integrated screening services and linkage to primary care

SUPPLEMENTAL DATA

Supplementary Figure S1: Screening and linkage cascade for hypertension. The grey box with bold border is the denominator for calculation of linkage cascade completion; grey boxes without bold borders all contribute the numerator.

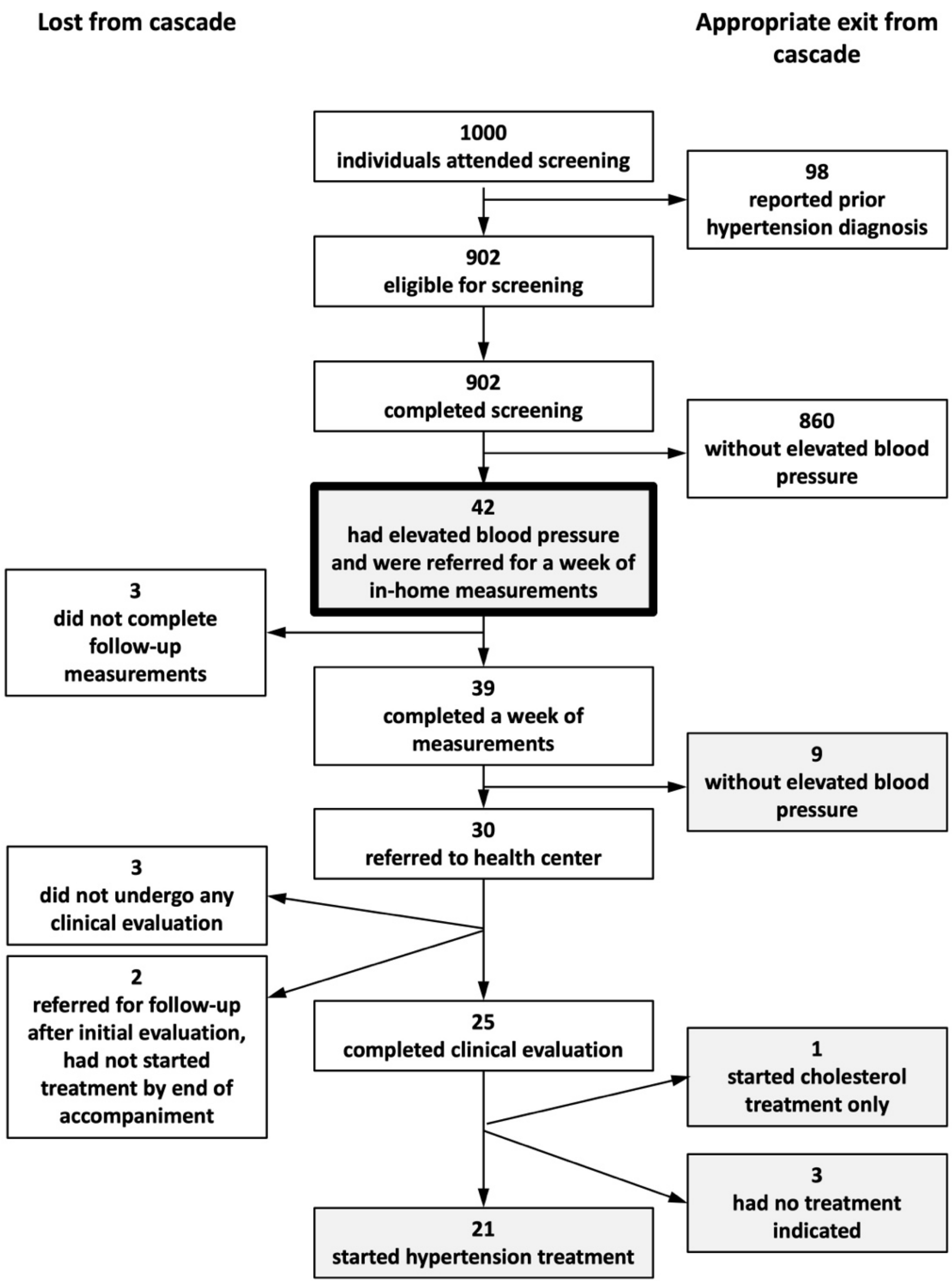

Supplementary Figure S2: Screening and linkage cascade for diabetes. The grey box with bold border is the denominator for calculation of linkage cascade completion; grey boxes without bold borders contribute to the numerator.

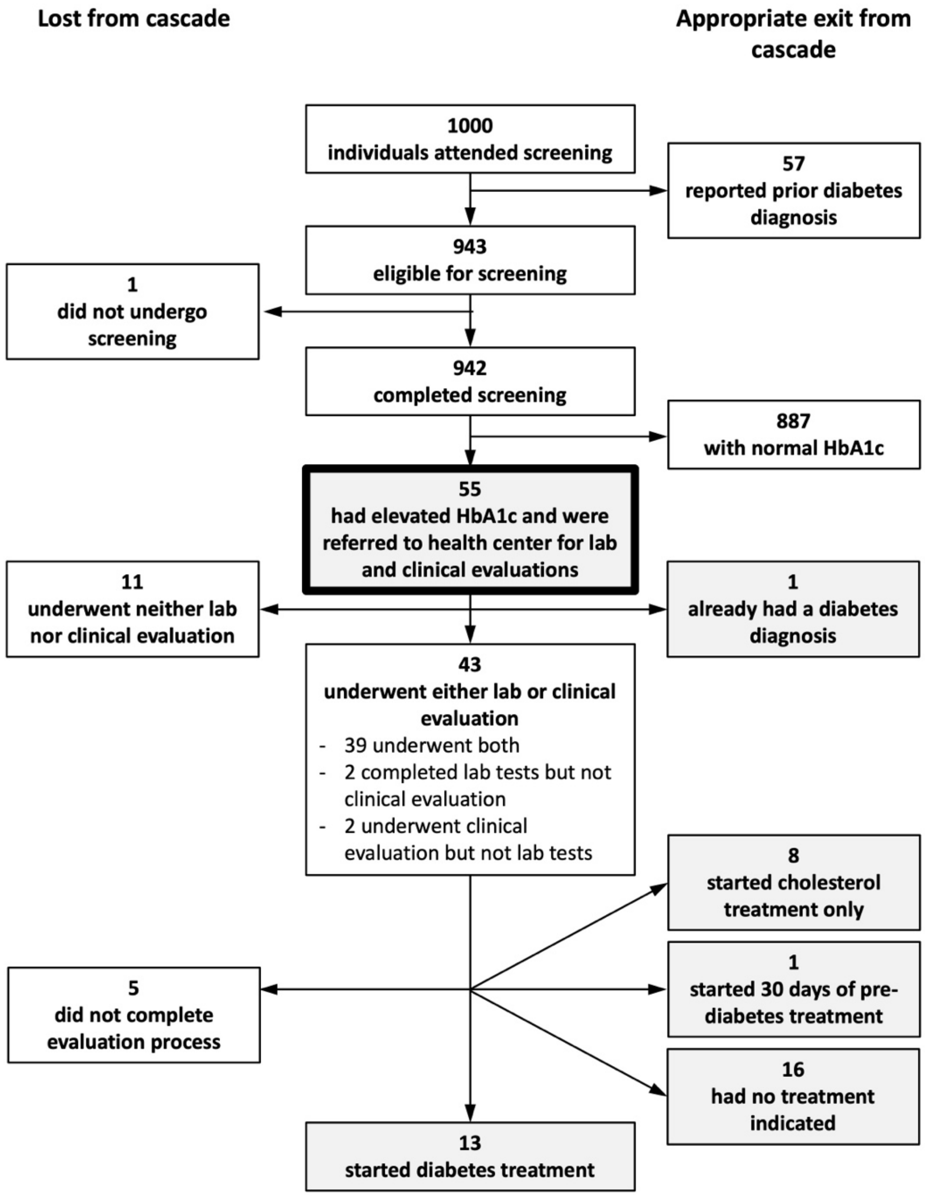

Supplementary Figure S3: Screening and linkage cascade for depression. The grey box with bold border is the denominator for calculation of linkage cascade completion; grey boxes without bold borders contribute to the numerator.

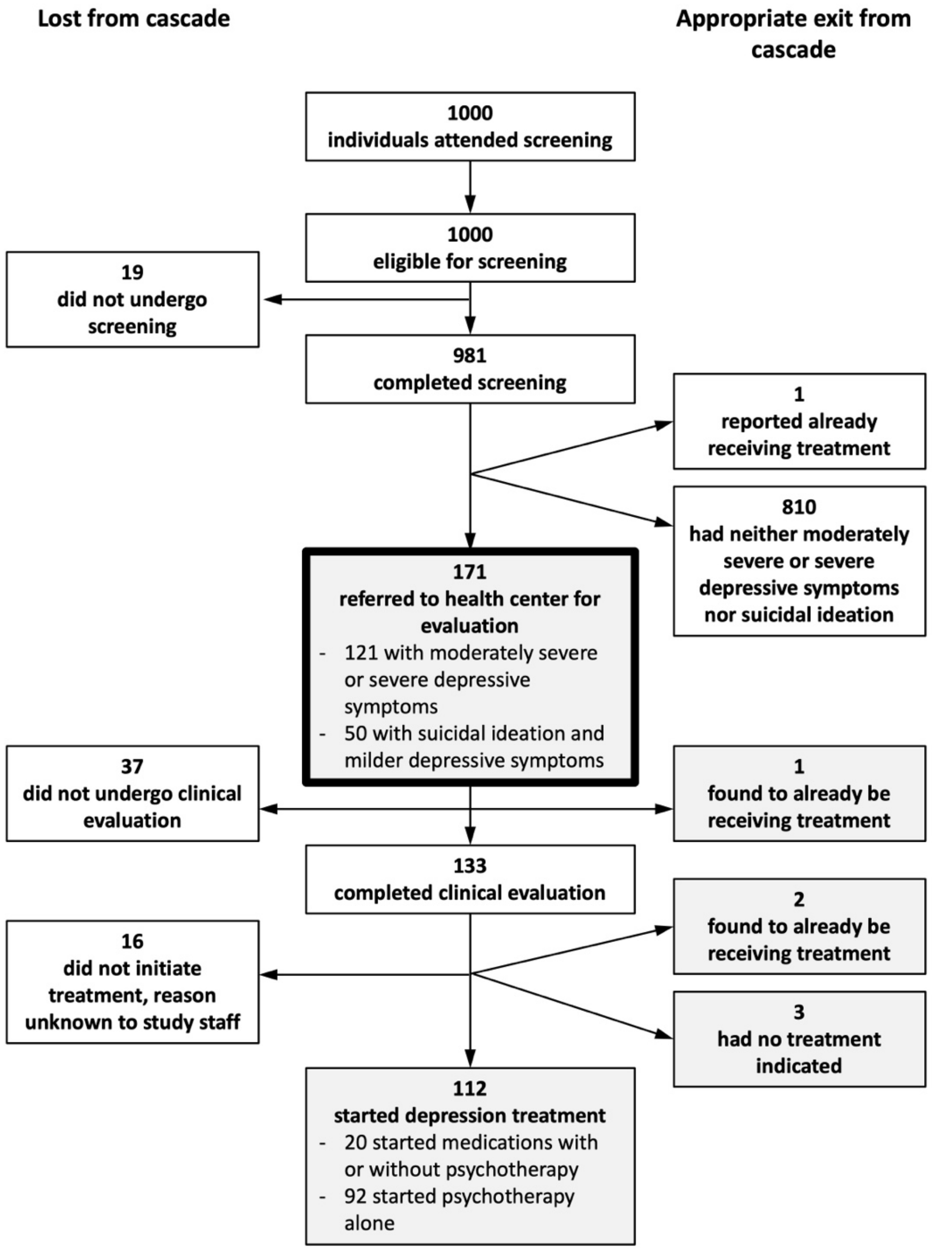

Supplementary qualitative data: Elaboration of barriers and facilitators to implementation

Barriers to implementing screening

Approachability

Given the limited number of individuals screened per campaign, it is challenging to equitably distribute the screening appointments among community members. Some attendees were informed of the campaign by their local community leaders, while others saw the campaign or a related flyer; other community members who may have benefitted from the campaign were not aware of it or could not attend for other reasons.

Acceptability

Despite all participants being required to pass through the mental health screening station, stigma around mental health led some individuals to not participate in the campaign or to leave mid-way through before the mental health station.

Availability and accommodation

Equipment and personnel limitations at various screening stations led to delays. Equipment limitations that led to increased wait times included the processing speed of the HbA1c machine, the number of available blood pressure cuffs, and the number of x-ray machines. Personnel limitations that led to delays included the number of available trained counselors and mental health screeners. At the mental health station in particular, delays often occurred as people became emotional and needed additional time for psychoeducation and support.

In general, there was a greater demand for screening among the community than the capacity of the campaign. As a result, some people were turned away from the campaign once it had reached capacity. We were also not able to evaluate participants with prior diagnoses of diabetes, hypertension or tuberculosis to prevent limiting space for participants without diagnoses.

Appropriateness

The PHQ-9 does not screen for mental health conditions aside from depression, so participants experiencing other significant challenges or stressors such as anxiety or interpersonal violence may not have been identified.

Other

Some participants did not complete all screening stations. Reasons for this included: needing to attend to an urgent matter, thinking the campaign would be shorter despite the expectations set during the consent process, not realizing that there were screenings remaining, and not wanting to go to the mental health station.

Barriers to linking participants to care

Approachability

Some participants, particularly older adults, forgot about appointments with community health workers and were confused about why they were being contacted. Participants who did not have personal cell phones or were traveling out of town were difficult to contact.

Acceptability

Lower acceptability of mental health care led to missed appointments. Some participants placed a lower relative priority on mental health than other conditions, and stigma—whether from participants' families who rejected mental health diagnoses or from participants' own self-stigma—reduced adherence. Participants also missed appointments for other health conditions due to competing priorities. Reasons included having busy work schedules, childcare needs, not having time, being out of town, forgetting about appointments, and frustration with needing to return to health centers multiple times before receiving a diagnosis.

The stigma of associating Socios En Salud's community health workers with tuberculosis led to the rejection of home visits. People did not want to be contacted by community health workers for other reasons as well, including feeling that it was a violation of their privacy or wanting to arrange health center appointments on their own.

Availability and accommodation

Provider shortages in health facilities extended wait times for appointments. Also, some health facilities lacked lab supplies and capacity, limiting the number of laboratory appointments available per day and requiring participants to arrive early in the morning to secure appointments. Reagent for glucose testing was not available in certain centers, leading to delays in diabetes diagnosis and necessitating private paid lab tests.

Participants experienced physical difficulties in accessing health facilities, ranging from mobility challenges to lengthy travel distances from their homes.

Inflexible health system protocols made services less convenient and timely. For example, a physical identification card was required to make appointments; appointments could only be made at EsSalud clinics at the beginning of each month; and participants undergoing evaluation for tuberculosis had to delay mental health appointments. Some psychologists and medical doctors would not accept and treat patients without official documentation of the campaign's diagnostic testing. Resistance from health center staff occurred when they were not aware of the influx of referrals from campaigns. CHWs at health centers were also resistant to external CHWs accompanying participants within their catchment area.

A complex and difficult to follow work flow for mental health evaluations lengthened time to diagnosis and increased the number of appointments participants had to attend. For example, after an initial evaluation at the community clinic, participants were often referred to a community mental health center for further evaluation, before ultimately being sent back to their community clinic for care—often without a clear diagnosis. Some participants had to wait a long time (weeks to months) for their community mental health center referrals to become available.

Lastly, siloing between health systems led participants to repeat blood tests in order to receive their diagnoses. Each health system required blood tests from their own laboratories, particularly EsSalud (employment-sponsored health insurance). The Ministry of Health centers (MINSA) only accepted external blood tests if they were ordered internally but were unable to be fulfilled at their facilities based on available laboratory supplies.

Affordability

The opportunity cost from missing work to attend a health center appointment was a barrier for participants, especially those who needed to request multiple days off for various appointments. Working outside the home, often on a variable schedule, also made it difficult for participants to have CHWs visit their homes for daily blood pressure readings.

Insurance issues reduced the affordability of attending health center appointments. Some participants were not enrolled in national health insurance, were temporarily uninsured due to changing employment, or were assigned to a clinic in a different geographic region than their current residence. Participants had delayed care waiting to enroll in insurance or switch their enrollment to the appropriate clinic site. Some participants could not afford to pay for lab tests or appointments privately when they were not otherwise available.

Appropriateness

Dissatisfaction with the treatment received at health facilities, such as connecting poorly with a psychologist, made participants hesitant to return for follow-up care.

CHWs who were specialized in accompaniment for tuberculosis lacked expertise in accompanying participants with other conditions, such as depression with suicidal ideation.

Other

CHWs experienced secondary mental health trauma from accompanying participants for mental health evaluations, especially when appointments were delayed and CHWs provided support to participants during an extended waiting period.

Facilitators of implementing screening

Approachability

Simultaneously offering screening for multiple conditions during the campaign appealed to a broader population, as people expressed interest in multiple conditions being screened. Also, in partnership with local community leaders, efforts were made to increase equity when disseminating information to potential attendees; for example, leaders sent out community-wide group chat messages and the first 30 individuals to respond were offered a place at the campaign.

Acceptability

Universal mental health screening for all participants reduced stigma around being seen at the mental health screening station. Additionally, setting clear expectations during the informed consent process around the anticipated duration of the campaign reduced early departures and dissatisfaction about wait times.

Availability and accommodation

Conducting campaigns in a wider geographic region than prior tuberculosis screening campaigns allowed for the identification of otherwise undetected cases.

Flexibility around the order of the screenings reduced the impact of equipment limitations on wait times. Two main approaches were taken to mitigate delays due to HbA1c machine processing time. First, some participants continued on to depression screening while the HbA1c machine was processing their sample, then a CHW accompanied the patient back to the prior station to receive their result. Second, some participants went to the hypertension screening station after the diabetes station, where they were required to sit for five minutes prior to having their blood pressure taken; this allowed time for their HbA1c to result.

Flexibility and modifications in personnel allocation improved flow and reduced wait times. Staff from the enrollment station was reallocated to the counseling station once enrollment was complete to increase the number of trained counselors available. The doctor was able to spend more time with participants during clinical evaluations relative to higher-volume tuberculosis

screening campaigns, allowing for thorough history taking and assessment. Lastly, the addition of a second screener to the depression station allowed more time for participants to converse and receive psychoeducation.

Appropriateness

The depression screening station was responsive to participants' varied needs. Some participants who did not meet referral criteria on the PHQ-9 for depression shared pertinent mental health challenges that would benefit from psychological evaluation and support; these participants were counseled about seeking care at their community health center. Referral for follow-up care was provided for participants experiencing violence at home, even if they did not meet depression referral criteria.

Other

CHWs were positioned in various locations around the campaign in order to direct participants between screening stations and help prevent participants from accidentally missing a screening station or leaving before they received counseling.

Facilitators of linking participants to care

Approachability

Involving participants' families in the accompaniment process helped facilitate linkage to care. For participants who had difficulty remembering the follow-up care that was arranged for them, the CHWs connected with family members to help remind and accompany participants to their appointments. For participants without reliable cell phone access, CHWs either arranged home visits for communication or connected over the phone with a trusted family member. If participants did not have reliable phone access to receive a phone-based mental health intervention, they were referred to a health center for psychology follow-up instead.

Acceptability

Participants were empowered to express their preferences on the level of support they desired from CHWs, increasing acceptability. For example, some participants requested to arrange their own appointments or to attend appointments unaccompanied, while others preferred more support. When participants did not have time for follow-up care, but were interested in receiving support in the future, a joint plan was made to establish a timeline for re-engagement from the CHW.

When family members expressed stigmatizing beliefs about mental health care, participants used documentation from the campaign to validate their experiences and explain the importance of pursuing follow-up care.

Availability and accommodation

Health care centers were advised about campaigns in advance, and certain providers were willing to organize the workflow in their health centers on campaign days to make the linkage to care process very efficient. For example, some providers programmed their health center shifts for the day of the campaign to be able to promptly take all referrals. In health centers with their own laboratories, blood tests could be performed the same day and diagnoses could be provided rapidly. Invested providers also conducted home visits for mental health evaluations of older

adults with mobility issues and other vulnerabilities rather than requiring them come to the health center.

Having contacts within the health systems expedited appointment scheduling and reduced wait times, especially in the EsSalud system where appointments could only be arranged during limited windows each month.

Once laboratory supply issues were identified, SES donated the necessary supplies through the Ministry of Health to those particular centers to improve access to timely care.

Creative solutions were devised to address health system inflexibilities. For example, CHWs received approval from health centers to schedule appointments with digital rather than physical identification cards. Also, the campaign's psychologist and doctor signed forms detailing the diagnostic tests performed in order to facilitate appointments with providers who required official documentation.

Affordability

CHWs helped address various financial barriers to linkage to care. For participants who were not enrolled in national health insurance or the appropriate clinic site, CHWs helped facilitate and expedite the enrollment process with the help of the SES social protection team. When laboratory supplies were not available for required tests or the cost of health center appointments was prohibitive, social support funds were obtained to pay for lab tests privately or to cover the appointment costs. Creative alternative solutions were also arranged, such as connecting participants with free-of-charge private mental health clinics when the cost of follow-up mental health care sessions was prohibitive. Lastly, social support funds were obtained for transportation costs when they posed a barrier for participants.

For participants with work schedules that limited their availability for at-home blood pressure measurements, CHWs made individualized plans around their work schedules.

Appropriateness

Health center appointments allowed participants to be evaluated for and diagnosed with other health conditions besides their presenting concerns. Participants became familiar with the locations and processes of their local health centers and laboratories, and they gained an increased understanding of their overall health.

In cases of TB diagnostic uncertainty, SES utilized their personnel infrastructure to send radiographs suspicious for tuberculosis to an affiliated physician for a second opinion.

When participants did not connect well with a care provider at their health center, the CHWs helped mitigate hesitations about returning for care with a different provider.

CHWs with extensive experience accompanying participants with TB received training to be effective multi-disease health system navigators. Specialized training for CHWs was also provided by the campaign's psychologist about addressing mental health stigma and navigating cases where participants experienced suicidal ideation—expanding the capacity of the CHWs.

Other

CHWs received trainings and support from the campaign's psychologist to address their secondary mental health trauma.

Health center staff was less resistant to the influx of referrals and external CHWs once they were provided with advance warning of the campaigns and an explanation of the CHWs' unique role and training.
